# A study on the correlation between the prognosis of HPV infection and lesion recurrence after cervical conization

**DOI:** 10.3389/fmicb.2023.1266254

**Published:** 2023-10-06

**Authors:** Junling Lu, Shichao Han, Ya Li, Jing Na, Jun Wang

**Affiliations:** Department of Gynecology and Obstetrics, Second Affiliated Hospital of Dalian Medical University, Dalian, China

**Keywords:** HPV infections, cervical lesions, cervical conization, recurrence, prognosis

## Abstract

**Introduction:**

Persistent human papillomavirus infection is an important factor in the development of cervical cancer, which is usually a long process evolving from the development of squamous intraepithelial lesions (SIL), also referred to as cervical intraepithelial neoplasia (CIN). Local treatment of advanced squamous intraepithelial lesions, also regarded as High-Grade Squamous Intraepithelial Lesion, may be effective in preventing cancer.

**Objective:**

To promptly identify high-risk patients with a tendency to recurrence.

**Methods:**

We retrospectively analyzed the clinical data of 300 patients with high-grade squamous intraepithelial lesions of the cervix admitted to the Second Affiliated Hospital of Dalian Medical University from 2019 to 2020 to investigate the relationship between recurrence of cervical lesions and postoperative regression of HPV infection, as well as other related risk factors.

**Results:**

We found that the HPV-negative rates were 81.81, 85.71, and 90.91% at 6, 12, and 24 months, respectively, and the average lesion recurrence rate was 8.16%, with a median time to recurrence of 14 months in patients undergoing CKC for HSIL. The risk of cervical squamous intraepithelial lesions was highest in patients with HPV16. Patients over 61 years of age had the lowest postoperative HPV-negative rate. The conversion rate was significantly lower in patients with multiple HPV genotypes than in those with single HPV infection (*p* < 0.05). The probability of recurrence was higher in patients with the same HPV infection genotype before and after surgery than in patients with different infection genotypes before and after surgery (*p* < 0.05).

**Conclusion:**

Combined with the literature review, we believe that patients aged ≥50 years, with ≥3 pregnancies and births, a history of smoking, and consistent genotypes of preoperative and postoperative HPV infection in cervical conization have more HPV re-infection or persistent infection, and that these factors may be high-risk factors for lesion recurrence. For patients with possible potential high-risk factors, we need to carry out individualized follow-up and focused management, take timely and effective management measures, optimize the treatment plan, reduce the recurrence rate, prevent HSIL and cervical cancer, improve the quality of patient’s survival, and improve the prognosis.

## Introduction

At present, cervical cancer has become a public health problem of global concern. It is one of the most common malignant tumors among women in the world, ranking fourth in incidence rate and mortality (Ferlay et al., 2015). High-risk Human Papilloma Virus (HPV) infection is considered the main cause of cervical cancer (Schiffman et al., 2007). Persistent human papilloma virus infection is an important factor in the development of cervical cancer, which is usually a long process evolving from the development of squamous intraepithelial lesions (SIL), also referred to as CIN. Local treatment of advanced squamous intraepithelial lesions, also regarded as High-Grade Squamous Intraepithelial Lesion (HSIL), may be effective in preventing cancer. So cervical cancer is the only malignant tumor that can be prevented by vaccination, and we can achieve early detection, treatment, and prevention through cervical precancerous screening. It is very important to detect cervical lesions in time and block their development. The American Society for Colposcopy and Cervical Pathology (ASCCP) recommends that cervical conization with fertility preservation be the first choice for HSIL with satisfactory Colposcopy examination. Cervical conization includes loop electrosurgical excision procedure (LEEP) and cold knife conization (CKC), but about 15% of patients experience varying degrees of residual or recurrence after surgery (Kocken et al., 2011). How to detect high-risk recurrence patients in time is still inconclusive. Therefore, this article attempts to analyze the prognosis of HSIL cervical conization surgery with HPV infection, explore the relationship between cervical lesion recurrence and HPV prognosis, and other related risk factors, with the aim of timely identify high-risk patients with recurrence tendency, and improve the survival rate and prognosis of patients.

## Methods

This article retrospectively analyzed the clinical data of 300 patients with high-grade squamous intraepithelial lesions (HSIL) of the cervix admitted to the Second Affiliated Hospital of Dalian Medical University from 2019 to 2020. The inclusion criteria for cases are those with HPV infection and pathologically diagnosed as HSIL through cervical biopsy. All patients underwent CKC for treatment. We collected age, pathological type, and involvement of glands in the lesion, HPV infection type and genotype, pregnancy and childbirth history, smoking history, postoperative HPV follow-up results at 6, 12, and 24 months, as well as the HPV infection status and lesion characteristics of recurrent patients. The follow-up period was until January 2023. All information is obtained by consulting medical records. The study was approved by the Ethics Committee of the Second Affiliated Hospital of Dalian Medical University. We used SPSS statistical software for data analysis, counting data use cases and percentage, and comparing multiple groups using χ^2^ tests or Fisher’s exact probability method, using Bonferroni correction for pairwise comparison. The difference with *p* < 0.05 is statistically significant.

## Results

Analysis of clinical data characteristics of patients with high-grade squamous intraepithelial lesions of the cervix.

Among the 300 patients we collected, the average age was 41.13 years (21–70 years). 245 patients completed follow-up, with an overall follow-up rate of 81.67%. 20 patients experienced recurrence within 4 years after surgery, with an average recurrence rate of 8.16% and a median recurrence time of 14 months. Postoperative pathology of 153 patients with cervical cone resection revealed gland involvement. There was no significant difference in the postoperative negative conversion rate between them and patients without gland involvement. We believe that whether the gland is involved or not has no significant effect on the postoperative recurrence rate (Table 1).

**Table 1:** Chi square analysis of gland involvement and HPV negative conversion rate.

Gland involvement	Negative	Positive	Total	$\chi^{2}$	*p*
Y(Yes)	118(77.12%)	35 (22.88%)	153	0.043	0.836
N(No)	72 (78.26%)	20 (21.74%)	92		
Total	190 (77.55%)	55 (22.45%)	245		

### Analysis of HPV infection genotypes before and after operation

Table 2 lists the statistics of HPV infection genotypes of HSIL patients before operation, and the top five are genotypes 16, 52, 58, 33, and 31 in turn. 146 cases of cervical high-grade squamous intraepithelial lesions caused by HPV16, accounting for 35.78%. The genotypes of HPV reinfection after cervical conization, and the top five are genotypes 16, 58, 52, 53, and 39. Thus it can be seen that the common HPV infection genotypes in patients with HSIL are 16, 52, and 58 genotypes. Genotypes 16 of HPV has the highest risk of cervical squamous intraepithelial lesions.

**Table 2:** Statistics of HPV infection genotypes before operation and reinfection genotypes after surgery.

Genotypes before operation	Cases	Percentage (%)	Reinfection genotypes after surgery	Cases	Percentage (%)
16	146	35.78	16	23	19.83
52	41	10.05	58	22	18.97
58	39	9.56	52	13	11.21
33	21	5.15	53	11	9.48
31	18	4.41	39	8	6.90
18	17	4.17	56	7	6.03
35	12	2.94	51	6	5.17
51	12	2.94	CP8304	6	5.17
53	12	2.94	33	5	4.31
68	11	2.70	59	4	3.45
39	10	2.45	68	4	3.45
56	8	1.96	66	3	2.59
66	7	1.72	11	1	0.86
81	7	1.72	31	1	0.86
Not genotyped	14	3.42	45	1	0.86
45	6	1.47	6	1	0.86
59	5	1.23			
6	4	0.98			
42	3	0.74			
82	3	0.74			
11	2	0.49			
Others	10	2.45			

Other genotypes include: 9, 26, 40, 43, 54, 55, 61, 73, 78, 83, etc.

### Analysis of age and recovery from HPV infection after cervical conization

**Figure 1:**
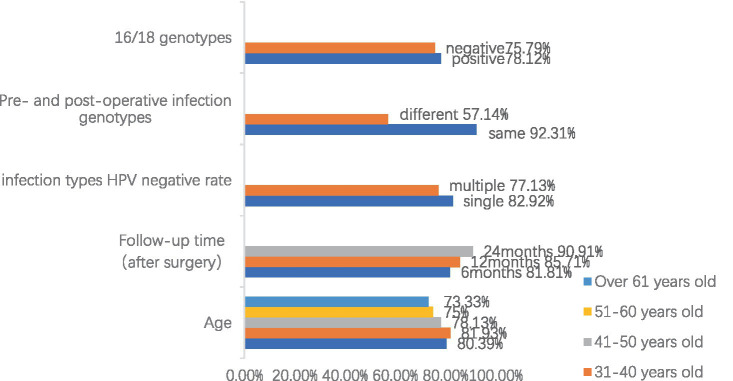
Summary of risk factors and negative rate of HPV after cervical conization (Due to the multiple categories of data, the legend only indicates different age groups, and the remaining data are marked as follows: negative = 16/18 genotypes negative, positive = 16/18 genotypes positive, same = the same genotype before and after surgery, different = different genotype before and after surgery, single = single HPV genotype infection, and multiple = multiple HPV genotypes infection.).

Figure 1 shows the statistics of HPV retesting after cervical conization in different age groups, and a total of 192 cases were screened with negative results of the last HPV test, with an overall HPV conversion rate of 79.18%. We found that patients over 61 years of age had the lowest postoperative HPV conversion rate of about 73.33%. The conversion rate was also lower in the age group of 51–60 years, at 74.70%, with little difference in the rate between the ages of 30 and 50 years. However, there was no significant difference between the conversion rates of each age group by χ^2^ test.

### Correlation of multiple HPV infections, HPV genotypes, and recovery from HPV infection after cervical conization

Of the 245 follow-up patients who underwent cervical conization for HSIL, 110 patients were retested for HPV at 6 months postoperatively with a negative rate of 81.81%, 98 patients were retested for HPV at 12 months postoperatively with a negative rate of 85.71%, and 66 patients were retested for HPV at 24 months postoperatively with a negative rate of 90.91%. We can see that the negative rate of HPV test is gradually increasing with time, and the negative rate of HPV test is more than 90% at 2 years after the operation. This also shows that cervical conization is effective in removing HPV. Meanwhile, we found that the negative rate of 49 patients with multiple HPV infections was 77.13%, which was significantly lower than those with HPV mono-infections (*p* = 0.049). We also statistically analyzed the sex-negative rate of patients infected with HPV genotypes 16 or 18 and found that whether they were infected with HPV genotypes 16 or 18 did not significantly affect the negative rate (*p* = 0.681).

For patients who were reinfected with HPV (first postoperative HPV-negative review and second or subsequent HPV-positive review), we sorted the data according to whether the patients were infected with the same HPV genotypes before and after surgery, and we analyzed the effect of the consistency of the preoperative and postoperative infection genotypes on the recurrence of lesions. Due to the small sample size and some subgroup sample counts less than 5, the use of the traditional chi-square test tends to bias the test results, so Fisher’s exact test was used for chi-square estimation. The results showed that at the 10% significance level, the probability of recurrence was higher in patients with the same HPV infection genotype before and after surgery than in patients with different infection genotypes before and after surgery.

### Correlation between the number of pregnancies and births and HPV infection and regression

We counted the maternal history of 271 patients and found that the number of pregnancies and births had no significant effect on the HPV conversion rate. Due to the small sample size and some subgroup sample counts less than 5, the use of the traditional chi-square test is prone to bias the test results, so Fisher’s exact test was used for chi-square analysis (Table 3).

**Table 3:** Chi-square test of the number of pregnancies, births, and HPV negativity rate.

Number of pregnancies	Negative	Positive	Total	$\chi^{2}$	*p*
0	33(86.84%)	5 (13.16%)	38		
1–2	131 (82.39%)	28 (17.61%)	159	0.743	0.878
3–4	89 (82.41%)	19 (17.59%)	108		
5 and more	11 (78.57%)	3 (21.43%)	14		
Number of births					
0	53 (84.13%)	10 (15.87%)	63		
1	171 (83.01%)	35 (16.99%)	206	0.090	0.977
2 and more	47 (82.46%)	10 (17.54%)	57		
Total	272 (83.18%)	55 (16.82%)	327		

## Discussion

### HPV and cervical squamous intraepithelial lesions

Persistent human papillomavirus infection is an important factor in the development of cervical cancer, which is usually a long process evolving from the development of squamous intraepithelial lesions (SIL), also known as cervical intraepithelial neoplasia (CIN). Early studies have shown that women with persistent HPV infection have a significantly increased risk of developing cervical squamous intraepithelial lesions, which can progress to cancer if not detected and treated in time (Walboomers et al., 1999; Bosch et al., 2002). In other words, local treatment of advanced squamous intraepithelial lesions, also known as HSIL, may be effective in preventing cancer (International Agency for Research on Cancer, 2005, 2020; Kyrgiou et al., 2020). Local cervical treatments include large loop excision of the transformation zone (LLETZ), cold knife conization (CKC), loop electrosurgical excision procedure (LEEP), or ablation. Because cervical conization allows assessment of the margins of the excised tissue for residual lesions, excisional treatment is currently considered superior to ablation (Perkins et al., 2020). Arbyn et al. showed that the average risk of recurrence after surgery for high-grade lesions was only 6.6%. Among them, the risk of recurrence of HSIL or CIN was 6.7% for LLETZ, while CKC or laser conization had only a 2.1–2.2% risk of recurrence (Arbyn et al., 2017). At the same time, several studies have shown that the recurrence rate after CKC ranges from 1.4 to 2.2% (Santesso et al., 2016; Arbyn et al., 2017; Zhang and Lin, 2022), while the failure rate after LEEP treatment ranges from 7 to 14% (Kreimer et al., 2006; Lubrano et al., 2012; Kuroki et al., 2016). All patients in our study underwent CKC, the recurrence rate of the lesions was 8.16%, the median time to lesion recurrence was 14 months, and the pathological type was LSIL. We believe that CKC and LEEP have their own advantages, and the treatment should be individualized according to the patient’s condition. Updates and improvements in surgical approaches need to be further explored and confirmed by multicenter, large-sample prospective studies.

### Analysis of HPV regression after cervical conization

There is an association between the onset, progression, treatment, and prognosis of SIL or CIN and HPV infection (Salvadó et al., 2021). However, HPV infection is asymptomatic in most patients and resolves spontaneously within 1–2 years (Frazer, 2009). The 2-year HPV-negative rate after cervical conization in our study was more than 90%. To reduce the psychological and economic burden on patients, we believe that a 6-month postoperative follow-up period is appropriate. In addition, Alonsol et al. and Perkins et al. (2020) showed that HPV viral load was also strongly related to postoperative residue or recurrence, with higher rates of postoperative residue or recurrence in the group of patients with high preoperative HPV viral load. Thus, persistent HPV infection, as well as HPV viral load, may be a high-risk factor for residue or recurrent postoperative lesions. Whether or not to include HPV viral load as one of the postoperative tests then needs to be supported by more clinical research data. Although surgery removes most of the HPV, it does not remove it completely, and if residual HPV-infected lesions remain after surgery, it may lead to recurrence of the lesions or even eventual progression to cervical cancer.

### Age

We counted the HPV testing after cervical conization in different age groups. And we found that there was a relationship between the age of patients and postoperative residual or recurrence. We found that postoperative HPV-negative rates decreased progressively with increasing age or late menopausal period, although there was no dramatic difference in postoperative negative rates between age groups, with the lowest postoperative HPV-negative rates found in patients over the age of 61 years. It has been shown (Bilibio et al., 2019) that low estrogen levels in postmenopausal women reduce the number of cells secreting interferon-γ and tumor necrosis factor--α, thereby decreasing immune reactivity, and have an accelerating effect on the release of pro-inflammatory cytokines, which in turn promotes lesion progression. Petorius et al. (2006) followed patients after CIN conization and found that the subsequent risk of CIN 3 or cancer increased with patient age. Therefore, we believe that for elderly patients, especially postmenopausal patients, considering that their lesions are mostly hidden in the cervical canal and their immune function is declining, as well as the poor adherence to postoperative follow-up in elderly patients, an individualized plan of treatment should be formulated, and close postoperative follow-up should be performed to detect recurrent cases in time and take appropriate management measures.

### Maternity and smoking

Early studies have shown that pregnancy-associated elevated levels of estrogen or growth hormone, such as Human Chorionic Gonadotropin (HCG), may increase the activity of HPV molecules, which in turn may influence the natural history of HPV infection and the progression of lesions (Nair et al., 2005; Delvenne et al., 2007). Jensen et al. (2013) found that childbirth increased the risk of HSIL even more than the risk of persistent HPV infection. The mechanism by which pregnancy influences the progression of HPV infection to clinical lesions may be related to the pregnancy-associated increase in HPV molecular activity due to elevated levels of estrogen or growth hormone, such as human chorionic gonadotropin (HCG). However, there are also studies that show no increased risk of HPV infection during pregnancy (Banura et al., 2008; Schmeink et al., 2012; Trottier et al., 2015). In our study, we counted the maternal history of conization patients and found that the number of pregnancies and births did not have a significant effect on the HPV conversion rate after conization. Therefore, we believe that further prospective studies with large samples are needed to confirm the effects of pregnancy and maternal history on HPV infection and postoperative recurrence.

Smoking has been shown to be an independent risk factor for HSIL in young women after the onset of frequent sexual activity (Collins et al., 2010). Several studies have shown (International Collaboration of Epidemiological Studies of Cervical Cancer et al., 2006; Koshiyama et al., 2019) that smokers have an increased risk of developing cervical cancer compared with nonsmokers, and that the risk of squamous cell carcinoma of smokers increases with the number of cigarettes smoked every day and with decreasing age of smoking initiation. Inamine et al. (2012) found that smoking induces high plasma expression of vascular endothelial growth factor C (VEGF-C) in patients with CIN and that VEGF-C plays a major role in cervical lesions in smoking patients. However, it has also been shown (Hui-Qiong et al., 2016) that there is no statistically dramatic difference in the comparison of postoperative recurrence between smoking and non-smoking patients. The smoking history in our study was only one person, and there are fewer large-sample studies on the effect of smoking on postoperative HPV regression or lesion recurrence, so we believe this requires more data collection and further exploration.

### Effect of postoperative re-infection with HPV subtype and type on recurrence

Multiple infections refer to the coexistence of two or more HPV subtypes in patients with cervical cancer, which may be dual, triple or even more. Reinfection with HPV after cervical conization remains a high-risk factor for lesion recurrence, and which subtypes and types of infection are more likely to cause recurrence needs to be further explored. Gök et al. (2007) and Bais et al. (2009) reported that women infected with HPV16 were significantly more likely to develop residual/recurrent CIN than women infected with other HR-HPV genotypes. However, because the study included a small number of residual cases, this does not confirm the correlation between HPV genotype infection and recurrence. Nam et al. (2009) conducted a study on recurrent cases with negative margins and found that pre-taper HPV16 infection was significantly associated with persistence of High Risk-Human Papilloma Virus (HR-HPV) after taper. Furthermore, it has also been reported (Söderlund-Strand et al., 2014; Kang and Kim, 2016) that HPV18 is more likely to be related to recurrence of high-grade CIN (*p* < 0.05). Vintermyr et al. (2014) reported that 95.9% of recurrent CIN2+ patients had persistent HR-HPV infection, of which 74.5% were HPV16 or HPV 18. More than 60% of recurrent CIN2+ occurred within 3 years of initial conization, indicating a high risk of recurrence in patients with persistent HR-HPV infection. In this study, HPV16, HPV52, and HPV58 were the top three infection genotypes in order before conization, and HPV16, HPV58, and HPV52 were the top three infection genotypes in order after conization. HPV16 was the genotype with the highest infection rate before and after conization, but we statistically found that whether or not we were infected with HPV genotypes 16/18 did not have a significant effect on the rate of conversions (*p* = 0.836). Meanwhile, in our study, the negative rate of 49 patients with multiple HPV infections after cervical conization was 77.13%, which was significantly lower than that of those infected with a single HPV genotype. And we found that the probability of recurrence in patients with the same genotype of HPV infection before and after surgery was higher than that in patients with different genotypes of infection before and after surgery. There are still some limitations since some patients in this study were lost to follow-up and the use of LSIL or more defined as residual/recurrent, which may have influenced the statistical results to be biased. Therefore, the relevance between HPV genotype infection and recurrence needs to be confirmed by further studies.

### Vaccination and lesion recurrence

Human Papilloma Virus (HPV) vaccination is a primary prevention of cervical cancer and significantly reduces the risk of developing cervical lesions and even cervical cancer. Some scholars believe that HPV vaccination may reduce the risk of recurrence in patients with CIN who undergo surgery (Kang et al., 2013; Ghelardi et al., 2018; Bartels et al., 2020; Jentschke et al., 2020; Lichter et al., 2020; Di Donato et al., 2021), but others believe that vaccination will not play a role in postoperative patients with CIN (Sand et al., 2020). It is worth noting that in analyzing previous observational studies, we should consider the surgical margins to assess more objectively the effect of HPV vaccination after excisional treatment. The mechanism of prophylaxis after CIN is unclear. Some studies have emphasized a possible cross-immunization effect for initial prophylaxis with vaccines against HPV-related diseases, but the effect on recurrent infections and whether it prevents reactivation of latent HPV infections is unclear (Ault, 2007; Jenkins, 2008; Gravitt and Winer, 2017). We believe that HPV vaccination may decrease the risk of postoperative recurrence in patients with HSIL, but more studies are needed to further confirm the mechanism of action for better clinical application.

## Conclusion

In summary, the chances of patients becoming negative for HPV after cervical conization are high, and to reduce the psychological and economic burden on patients, we believe that a postoperative follow-up period of 6 months is appropriate. Whereas the numerous high-risk factors for HPV reinfection or persistent infection are inconclusive. In conjunction with the literature review, we believe that HPV reinfection or persistent infection was more common in patients aged ≥50 years, with ≥3 pregnancies and deliveries, a history of smoking, and consistent genotypes of pre- and postoperative HPV infection in cervical conization, all of which may be high-risk factors for lesion recurrence. For patients with potential high-risk factors, we need to provide individualized follow-up and targeted management, take timely and effective action, optimize treatment plans, reduce recurrence rates, prevent HSIL and cervical cancer, improve the quality of patient survival, and improve prognosis.

## Data availability statement

The original contributions presented in the study are included in the article/supplementary material, further inquiries can be directed to the corresponding authors.

## Ethics statement

The studies involving humans were approved by the Ethics Committee of the Second Affiliated Hospital of Dalian Medical University. The studies were conducted in accordance with the local legislation and institutional requirements. The participants provided their written informed consent to participate in this study.

## Author contributions

JL: Investigation, Writing – original draft, Writing – review & editing. SH: Data curation, Writing – original draft. JN: Methodology, Writing – review & editing. JW: Project administration, Supervision, Writing – review & editing. YL: Investigation, Writing – original draft.

## References

[ref1] Arbyn M. Redman C. W. E. Verdoodt F. Kyrgiou M. Tzafetas M. Ghaem-Maghami S.. (2017). Incomplete excision of cervical precancer as a predictor of treatment failure: a systematic review and meta-analysis. Lancet Oncol. 18, 1665–1679. doi: 10.1016/S1470-2045(17)30700-3, PMID: 29126708

[ref2] Ault K. A. (2007). Human papillomavirus vaccines and the potential for cross-protection between related HPV types. Gynecol. Oncol. 107, S31–S33. doi: 10.1016/j.ygyno.2007.08.059 18499916

[ref3] Bais A. G. Eijkemans M. J. C. Rebolj M. Snijders P. J. F. Verheijen R. H. M. van Ballegooijen M.. (2009). Post-treatment CIN: randomized clinical trial using hrHPV testing for prediction of residual/recurrent disease. Int. J. Cancer 124, 889–895. doi: 10.1002/ijc.23824, PMID: 19048594

[ref4] Banura C. Franceschi S. van Doorn L. J. Arslan A. Kleter B. Wabwire-Mangen F.. (2008). Prevalence, incidence and clearance of human papillomavirus infection among young primiparous pregnant women in Kampala, Uganda. Int. J. Cancer 123, 2180–2187. doi: 10.1002/ijc.23762, PMID: 18711697

[ref5] Bartels H. C. Postle J. Rogers A. C. Brennan D. (2020). Prophylactic human papillomavirus vaccination to prevent recurrence of cervical intraepithelial neoplasia: a meta-analysis. Int. J. Gynecol. Cancer 30, 777–782. doi: 10.1136/ijgc-2020-001197 32276936

[ref6] Bilibio J. P. Monego H. I. Binda M. L. A. dos Reis R. (2019). Menopausal status is associated with a high risk for residual disease after cervical conization with positive margins. PLoS One 14:e0217562. doi: 10.1371/journal.pone.0217562 31163055 PMC6548378

[ref7] Bosch F. X. Lorincz A. Munoz N. Meijer C. J. L. M. Shah K. V. (2002). The causal relation between human papillomavirus and cervical cancer. J. Clin. Pathol. 55, 244–265. doi: 10.1136/jcp.55.4.244, PMID: 11919208 PMC1769629

[ref8] Collins S. Rollason T. P. Young L. S. Woodman C. B. J. (2010). Cigarette smoking is an inde-pendent risk factor for cervical intraepithelial neoplasia in young women: a longitudinal study. Eur. J. Cancer 46, 405–411. doi: 10.1016/j.ejca.2009.09.015, PMID: 19819687 PMC2808403

[ref9] Delvenne P. Herman L. Kholod N. Caberg J. H. Herfs M. Boniver J.. (2007). Role of hormone cofactors in the human papillomavirus-induced carcinogenesis of the uterine cervix. Mol. Cell. Endocrinol. 264, 1–5. doi: 10.1016/j.mce.2006.10.014, PMID: 17145130

[ref10] Di Donato V. Caruso G. Petrillo M. Kontopantelis E. Palaia I. Perniola G.. (2021). Adjuvant HPV vaccinationto prevent recurrent cervical dysplasia after surgical treatment: a meta-analysis. Vaccine 9:410. doi: 10.3390/vaccines9050410 PMC8143003 33919003

[ref11] Ferlay J. Soerjomataram I. Dikshit R. Eser S. Mathers C. Rebelo M.. (2015). Cancer incidence and mortality worldwide: sources, methods, and major patterns in GLOBOCAN 2012. Int. J. Cancer 136, E359–E386. doi: 10.1002/ijc.29210, PMID: 25220842

[ref12] Frazer I. H. (2009). Interaction of human papillomaviruses with the host immune system: a well evolved relationship. Virology 384, 410–414. doi: 10.1016/j.virol.2008.10.004, PMID: 18986661

[ref13] Ghelardi A. Parazzini F. Martella F. Pieralli A. Bay P. Tonetti A.. (2018). SPERANZA project: HPV vaccination after treatment for CIN2+. Gynecol. Oncol. 151, 229–234. doi: 10.1016/j.ygyno.2018.08.033, PMID: 30197061

[ref14] Gök M. Coupé V. M. H. Berkhof J. Verheijen R. H. M. Helmerhorst T. J. M. Hogewoning C. J. A.. (2007). HPV 16 and increased risk of recurrence after treatment for CIN. Gynecol. Oncol. 104, 273–275. doi: 10.1016/j.ygyno.2006.10.011, PMID: 17157365

[ref15] Gravitt P. E. Winer R. L. (2017). Natural history of HPV infection across the lifespan: role of viral latency. Viruses 9:267. doi: 10.3390/v9100267 28934151 PMC5691619

[ref16] Hui-Qiong L. Xiu-Hua G. Cui Y. (2016). Analysis of factors associated with regression after LEEP treatment for cervical intraepithelial neoplasia. Mod. Hosp. 16, 489–491.doi: 10.3969/j.issn.1671-332X.2016.04.008

[ref17] Inamine M. Nagai Y. Mitsuhashi A. Nagase S. Yaegashi N. Yoshikawa H.. (2012). Cigarette smoke stimulates VEGF-C expression in cervical intraepithelial neoplasia (CIN) 1 and 2 lesions. Int. J. Clin. Oncol. 17, 498–504. doi: 10.1007/s10147-011-0322-3 21947598

[ref18] International Agency for Research on Cancer. (2005). Cervix Cancer Screening: IARC Handbooks of Cancer Prevention. Lyon: IARC Press, 1–302.

[ref19] International Agency for Research on Cancer. (2020). WHO Classification of Tumours: Female Genital Tumours. 5th Edn. Lyon: IARC Press.

[ref20] International Collaboration of Epidemiological Studies of Cervical Cancer Appleby P. Beral V. de González A. B. Colin D. Franceschi S.. Carcinoma of the cervix and tobacco smoking: collaborative reanalysis of individual data on 13,541 women with carcinoma of the cervix and 23,017 women without carcinoma of the cervix from 23 epidemiological studies. Int. J. Cancer (2006). 118: 1481–1495. doi: 10.1002/ijc.21493 16206285

[ref21] Jenkins D. (2008). A review of cross-protection against oncogenic HPV by an HPV-16/18 AS04-adjuvanted cervical cancer vaccine: importance of virological and clinical endpoints and implications for mass vaccination in cervical cancer prevention. Gynecol. Oncol. 110, S18–S25. doi: 10.1016/j.ygyno.2008.06.027, PMID: 18653221

[ref22] Jensen K. E. Schmiedel S. Norrild B. Frederiksen K. Iftner T. Kjaer S. K. (2013). Parity as a cofactor for high-grade cervical disease among women with persistent human papillomavirus infection: a 13-year follow-up. Br. J. Cancer 108, 234–239. doi: 10.1038/bjc.2012.513, PMID: 23169283 PMC3553518

[ref23] Jentschke M. Kampers J. Becker J. Sibbertsen P. Hillemanns P. (2020). Prophylactic HPV vaccination after conization: a systematic review and meta-analysis. Vaccine 38, 6402–6409. doi: 10.1016/j.vaccine.2020.07.055, PMID: 32762871

[ref24] Kang W. D. Choi H. S. Kim S. M. (2013). Is vaccination with quadrivalent HPV vaccine after loop electrosurgical excision procedure effective in preventing recurrence in patients with high-grade cervical intraepithelial neoplasia (CIN2-3)? Gynecol. Oncol. 130, 264–268. doi: 10.1016/j.ygyno.2013.04.050, PMID: 23623831

[ref25] Kang W. D. Kim S. M. (2016). Human papillomavirus genotyping as a reliable prognostic marker of recurrence after loop electrosurgical excision procedure for high-grade cervical intraepithelial neoplasia (CIN2-3) especially in postmenopausal women. Menopause 23, 81–86. doi: 10.1097/GME.0000000000000488 26057824

[ref26] Kocken M. Helmerhorst T. J. Berkhof J. Louwers J. A. Nobbenhuis M. A. E. Bais A. G.. (2011). Risk of recurrent high-grade cervical intraepithelial neoplasia after successful treatment: a longterm multi-cohort study. Lancet Oncol. 12, 441–450. doi: 10.1016/S1470-2045(11)70078-X, PMID: 21530398

[ref27] Koshiyama M. Nakagawa M. Ono A. (2019). The preventive effect of dietary antioxidants against cervical Cancer versus the promotive effect of tobacco smoking. Health 7:162. doi: 10.3390/healthcare7040162, PMID: 31847279 PMC6955726

[ref28] Kreimer A. R. Guido R. S. Solomon D. Schiffman M. Wacholder S. Jeronimo J.. (2006). Human papillomavirus testing following loop electrosurgical excision procedure identifies women at risk for posttreatment cervical intraepithelial neoplasia grade 2 or 3 disease. Cancer Epidemiol. Biomark. Prev. 15, 908–914. doi: 10.1158/1055-9965.EPI-05-0845 16702369

[ref29] Kuroki L. M. James-Nywening L. Wu N. Liu J. Powell M. A. Thaker P. H.. (2016). High-grade cervical dysplasia after negative loop electrosurgical excision procedure. J. Low. Genit. Tract Dis. 20, 300–306. doi: 10.1097/LGT.0000000000000260, PMID: 27575575 PMC5037026

[ref30] Kyrgiou M. Arbyn M. Bergeron C. Bosch F. X. Dillner J. Jit M.. (2020). Cervical screening: ESGO-EFC position paper of the European Society of Gynaecologic Oncology (ESGO) and the European Federation of Colposcopy (EFC). Br. J. Cancer 123, 510–517. doi: 10.1038/s41416-020-0920-9, PMID: 32507855 PMC7434873

[ref31] Lichter K. Krause D. Xu J. Tsai S. H. L. Hage C. Weston E.. (2020). Adjuvant human papillomavirus vaccine to reduce recurrent cervical dysplasia in unvaccinated women: a systematic review and meta-analysis. Obstet. Gynecol. 135, 1070–1083. doi: 10.1097/AOG.0000000000003833, PMID: 32282601

[ref32] Lubrano A. Medina N. Benito V. Arencibia O. Falcón J. M. Leon L.. (2012). Follow-up after LLETZ: a study of 682 cases of CIN-CIN3 in a single institution. Eur. J. Obstet. Gynecol. Reprod. Biol. 161, 71–74. doi: 10.1016/j.ejogrb.2011.11.023, PMID: 22177836

[ref34] Nair H. B. Luthra R. Kirma N. Liu Y. G. Flowers L. Evans D.. (2005). Induction of aromatase expression in cervical carcinomas: effects of endogenous estrogen on cervical cancer cell proliferation. Cancer Res. 65, 11164–11173. doi: 10.1158/0008-5472.CAN-05-1087, PMID: 16322267

[ref35] Nam K. Chung S. Kim J. Jeon S. Bae D. (2009). Factors associated with HPV persistence after conization in patients with negative margins. J. Gynecol. Oncol. 20, 91–95. doi: 10.3802/jgo.2009.20.2.91 19590719 PMC2705006

[ref37] Perkins R. B. Guido R. S. Castle P. E. Chelmow D. Einstein M. H. Garcia F.. (2020). 2019 ASCCP risk-based management consensus guidelines for abnormal cervical cancer screening tests and cancer precursors. J. Low. Genit. Tract Dis. 24, 102–131. doi: 10.1097/LGT.0000000000000525, PMID: 32243307 PMC7147428

[ref38] Petorius R. G. Peterson P. Azizi F. Burchette R. J. (2006). Subsequent risk and preaentation of cervical intraepithelial neoplasia (CIN3) or cancer after a colposcopic diagnosis of CIN1 or less. Am. J. Obstet. Gynecol. 195, 1260–1265. doi: 10.1016/j.ajog.2006.07.036 17074547

[ref39] Salvadó A. Miralpeix E. Solé-Sedeno J. M. Kanjou N. Lloveras B. Duran X.. (2021). Predictor factors for conservative management of cervical intraepithelial neoplasia grade 2:cytology and HPV genotyping. Gynecol. Oncol. 162, 569–574. doi: 10.1016/j.ygyno.2021.06.019, PMID: 34226019

[ref40] Sand F. L. Kjaer S. K. Frederiksen K. Dehlendorff C. (2020). Risk of cervical intraepithelial neoplasia grade 2 or worse after conization in relation to HPV vaccination status. Int. J. Cancer 147, 641–647. doi: 10.1002/ijc.32752 31648368

[ref41] Santesso N. Mustafa R. A. Wiercioch W. Kehar R. Gandhi S. Chen Y.. (2016). Systematic reviews and meta-analyses of benefits and harms of cryotherapy, LEEP, and cold knife conization to treat cervical intraepithelial neoplasia. J. Gynaecol. Obstet. 132, 266–271. doi: 10.1016/j.ijgo.2015.07.026 26643302

[ref42] Schiffman M. Castle P. E. Jeronimo J. Rodriguez A. C. Wacholder S. (2007). Human papillomavirus and cervical cancer. Lancet 370, 890–907. doi: 10.1016/S0140-6736(07)61416-0 17826171

[ref43] Schmeink C. E. Melchers W. J. Hendriks J. C. Quint W. G. Massuger L. F. Bekkers R. L. (2012). Human papillomavirus detection in pregnant women: a prospective matched cohort study. J. Women’s Health (Larchmt) 21, 1295–1301. doi: 10.1089/jwh.2012.3502, PMID: 23210493

[ref44] Söderlund-Strand A. Kjellberg L. Dillner J. (2014). Human papillomavirus type-specific persistence and recurrence after treatment for cervical dysplasia. J. Med. Virol. 86, 634–641. doi: 10.1002/jmv.23806 24123176

[ref45] Trottier H. Mayrand M.-H. Baggio M. L. Galan L. Ferenczy A. Villa L. L.. (2015). Risk of human papillomavirus (HPV) infection and cervical neoplasia after pregnancy. BMC Pregnancy Childbirth 15:244. doi: 10.1186/s12884-015-0675-0, PMID: 26446835 PMC4597450

[ref46] Vintermyr O. K. Iversen O. Thoresen S. Quint W. Molijn A. de Souza S.. (2014). Recurrent high-grade cervical lesion after primary conization is associated with persistent human papillomavirus infection in Norway. Gynecol. Oncol. 133, 159–166. doi: 10.1016/j.ygyno.2014.03.004 24631451

[ref47] Walboomers J. M. M. Jacobs M. V. Manos M. M. Bosch F. X. Kummer J. A. Shah K. V.. (1999). Human papillomavirus is a necessary cause of invasive cervical cancer worldwide. J. Pathol. 189, 12–19. doi: 10.1002/(SICI)1096-9896(199909)189:1<12::AID-PATH431>3.0.CO;2-F 10451482

[ref48] Zhang W. Lin Y. (2022). Modified method of cervical conization with hybrid use of a cold knife and an electric knife for high-grade squamous intraepithelial lesions. J. Int. Med. Res. 50:030006052211064. doi: 10.1177/03000605221106414, PMID: 35726589 PMC9218449

